# Stability of referential signalling across time and locations: testing alarm calls of Australian magpies (*Gymnorhina tibicen*) in urban and rural Australia and in Fiji

**DOI:** 10.7717/peerj.112

**Published:** 2013-07-23

**Authors:** Gisela Kaplan, Lesley J. Rogers

**Affiliations:** Centre for Neuroscience and Animal Behaviour, University of New England, Australia

**Keywords:** Alarm calls, Australian magpies, Signal stability, Australia, Fiji, Referential signals

## Abstract

In many avian species, vocal repertoire expands and changes throughout life as new syllables are added and sounds adapted to neighbours and circumstances. Referential signals, on the other hand, demand stability and lack of variation so that their meaning can be understood by conspecifics at all times. It is not known how stable such signals may be when the context is changed entirely but the point of reference remains unchanged. We investigated these questions in a rare case of forced translocation of an avian species, the Australian magpie (*Gymnorhina tibicen*), from Australia to the remote Fijian island of Taveuni decades ago. By using playbacks of vocalisations to 45 magpie groups in Australia, we first established that magpies use functionally referential signals in their alarm call repertoire signalling aerial danger (measured as looking up in response to a specific alarm call even though the speakers were on the ground). With these results in hand, we then used the same playbacks to magpie groups on the island of Taveuni. Our results showed that the meaning of one specific call (eagle alarm call) is stable and maintained even in populations that have been isolated from Australian conspecifics over many (at least 10) generations. To our knowledge, this is the first time such a stability of a referential signal has been shown in the natural habitat.

## Introduction

Alarm calls in vertebrates have been studied extensively and have been shown to be of remarkable consistency and context specificity, particularly in close-knit social groups. Among the most extensively described functionally referential alarm calls have been those of vervet monkeys (Seyfarth, Cheney & Marler, 1980), meerkats (Manser, Bell & Fletcher, 2001), prairie dogs (Kiriazis & Slobodchikoff, 2006) and ground squirrels (Swaisgood, Rowe & Owings, 2003). Functionally referential alarm calls and food-related calls have also been shown in dogs (Faragó et al., 2010), and in diverse avian species including domestic chickens (Evans, Evans & Marler, 1993; Wilson & Evans, 2012), ravens (Bugnyar, Kijne & Kotrschal, 2001), Siberian jays (Griesser, 2008) and Japanese great tits (Suzuki, 2012). It is far less clear whether groups isolated from one another and in different geographic regions maintain the same characteristics and functions of specific calls. We know even less of the strength of referential signals in a species when a group has been geographically isolated from any other groups of conspecifics over many generations. It is as yet rather unexplored whether specific behavioural patterns remain robust or are so changed (e.g., by learning or failure to learn in the new environment) that the communicable and functional link with the original population is lost.

**Figure 1:**
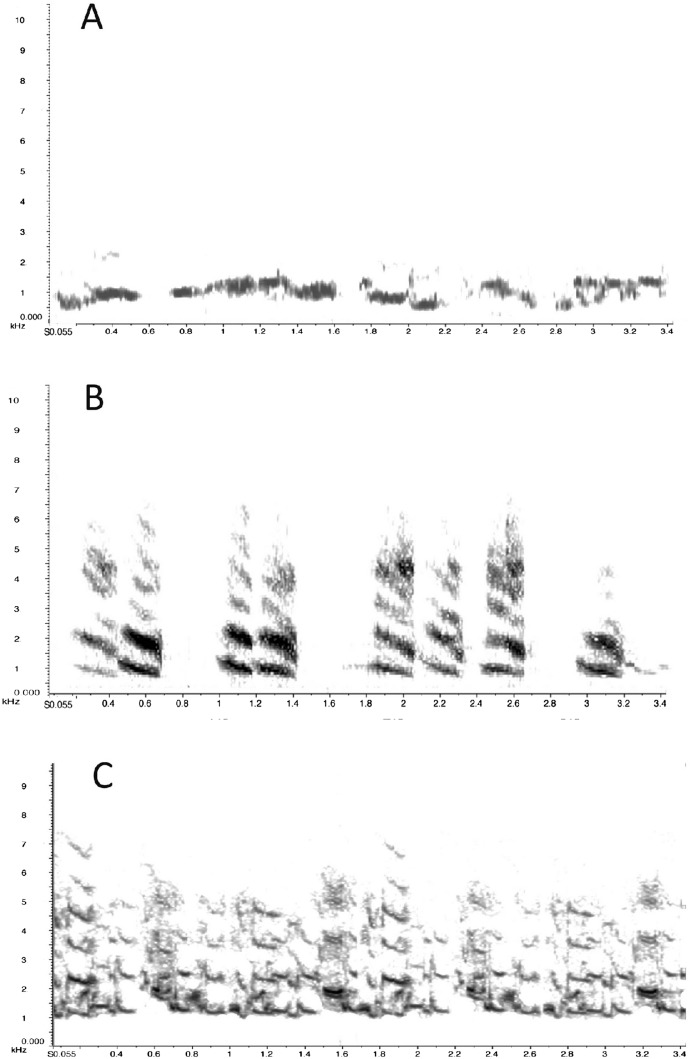
Sonograms of the calls presented to the magpies. Sonograms of the first 3.5 s of the five-minute playbacks are shown, enhanced to reveal details of the sounds used. As can be seen from the sonograms all three sounds are substantially different in structure and to the ear. (A) warble call, (B) generic alarm call, (C) the “eagle” alarm call. One playback consisted of a mixture of B and C in random sequence (see text for details).

The Australian magpie (*Gymnorhina tibicen*) is an ideal model species for examining such questions. Its widespread distribution across the Australian continent in different subspecies (Schodde & Mason, 1999), its territoriality circumscribing patterns of dispersal and its extremely large vocal repertoire (Kaplan, 2006a), also in alarm calling (Brown & Farabaugh, 1991; Kaplan, 2004), suggest opportunities for great variability in social communication and in alarm calls. The magpie’s main vocal expression, as one of the most versatile songbirds, consists of a song type referred to as warbling. The repertoire is generally very large (up to 893 syllables according to Farabaugh, Brown & Veltman, 1988) and extremely diverse and, at times, contains mimicry (Brown, Farabaugh & Veltman, 1988; Kaplan, 1999; Kaplan, 2006b). The warble, soft and undulating in sound, consists largely of pure tones ranging from approximately 1 to 2.5 kHz as well as some more complex harmonics (see Fig. 1A) and, because of a series of minibreaths and variations in membrane activation of the syrinx (Suthers, Wild & Kaplan, 2011) can be performed for hours. The magpie’s repertoire also includes many alarm calls that we described and classified previously (Kaplan et al., 2009). They range from a monosyllabic, harsh call that appears to signal the bird’s state of arousal to very complex and tonally distinct calls, produced when a raptor is seen in contexts in which such predators are a serious threat to adult birds (Kaplan et al., 2009). We had elicited these calls by presenting taxidermic models of raptors to a large number of magpie groups in rural and urban settings (Kaplan et al., 2009) and, as an extension of this research, can now present evidence of their function and use in entirely different locations, two in which magpies are native birds and abundant (inland New South Wales), and a third to which they had been introduced from New South Wales to a Pacific island.

Australian magpies were exported intentionally from Australia to New Zealand and to some Fijian islands with the express purpose of engaging them in biological control of insect pests that threatened to destroy local crops. In Fiji, this experiment failed dismally for the simplest of reasons. The stick insects that attack coconut palms in Taveuni and other Fijian islands live in the palm fronds high above the ground while magpies are strictly ground feeders and do not feed when roosting in trees.

According to the Clunie (1999), the first introduction (of white- and black-backed magpies) to the island of Taveuni happened as early as the 1880s. Subsequent introductions occurred in 1916 and in the 1930s and 1950s (Adrian Tarte, pers. comm., 2006). According to Adrian Tarte, the current owner of the estate to which the introductions were made, each time, only a single pair or a few magpies were released. The latest, so he remembered, apparently came from Taronga Zoo and were black-backed magpies. Although the attempt at biological control was completely unsuccessful, the magpies survived and bred on the island, albeit rather tenuously. As we observed, Pacific harriers (*Circus approximans*) and Fiji Goshawks (*Accipiter rufitorques*) are common and even plentiful in their territories. Peregrine falcons (*Falco peregrinus*) are less common but also present (Clunie, 1976). Compared to the Australian terrain in which we conducted our research on magpies, raptors were several times more abundant on Taveuni than in New South Wales and were an obvious presence in every environment in which we tested magpies on Taveuni - on plantations, near the shore and in wooded areas (judging by transect counts taken: 3 per 1 km^2^ in Taveuni, 2 per 10 km^2^ in inland New South Wales). They were an ever-present risk to both juvenile and adult magpies and we witnessed several foiled ambush attacks on juveniles by goshawks and harriers alike.

The experiments reported here tested the magpies’ responses to hearing two types of alarm call that we had recorded near Armidale in New South Wales (30°32′ S, 151°40′ E), where magpies (*G. tibicen*) are abundant (Barrett et al., 2003). One was the so-called generic alarm call (identified as Call B in our research of classifying alarm calls in magpies; see Kaplan et al., 2009 and Fig. 1B) used in a variety of low threat intensity alarm contexts and the tonally complex “eagle” alarm call (named ‘Call E’ in our earlier report; see Kaplan et al., 2009 and Fig. 1C) that is typically of much longer duration than the generic call (Kaplan et al., 2009). We presented these two calls alone and also in combination since magpies may produce both calls on sighting an eagle (Kaplan et al., 2009). Since warbling is not known to have any role in alarm calling, we presented it for comparison to the alarm calls (Fig. 1).

Our aim was twofold. In the first instance, it was important to play back specific alarm calls to confirm that the alarm calls suspected to be referential in earlier experiments were so consistently and that they triggered the same pattern of responses each time. The second aim, possible only if the first was shown to be correct, was to ascertain the stability of these calls by testing them in an isolated population of magpies. The question was whether the magpies on Taveuni would also spontaneously respond to alarm calls in a manner similar to or identical with those consistently observed in magpies in Australia, and do so by differentiating between the generic and “eagle” alarm call. Magpies are not known to have specific regional dialects and hence we hypothesised that both the generic and the eagle alarm call should be recognised. It was, of course, possible that long isolation had an influence on the meaning of these calls or they had been changed or lost in the birds translocated to Fiji.

Our scoring of the behavioural responses to presentation of these calls included the eye used to scan overhead since previous research had shown that birds and other species use the left eye to view predators. Domestic chickens show a left eye preference to look overhead when they hear their particular alarm call for aerial predators (Evans, Evans & Marler, 1993) and use their left eye to view the silhouette of a predator moving overhead (Dharmaretnam & Rogers, 2005). They also respond more readily to predators seen with the left eye (Rogers, 2000; Rogers, 2011). Since inputs from each eye are processed largely in the opposite hemisphere (Rogers & Kaplan, 2006; Rogers, 2008), this means that they used the left, lateral monocular field and their right hemisphere. Indeed, use of the right hemisphere in emergency situations to respond to novel stimuli and predators has been found in a number of vertebrate species (Lippolis et al., 2005; Lippolis et al., 2002; Koboroff, Kaplan & Rogers, 2008; summarised by MacNeilage, Rogers & Vallortigara, 2009). Eye preference is thus an important behavioural indicator of how certain stimuli are processed and was therefore included as a measure in our investigation of responses by magpies to presentation of alarm calls.

## Methods

### Experiments conducted in Australia

#### Animals tested

The magpies tested in Australia were the black-backed subspecies, *Gymnhorina tibicen tibicen*, of Eastern Australia (Schodde & Mason, 1999). Ten groups of magpies in different locations were tested. Mean group size was 4 (range 3–7) birds. The speaker used to present the calls was placed well within the boundaries of each group’s territory, determined prior to commencing the tests. The tests were conducted in localities 5–50 km from Armidale, a small town in NSW, Australia, (30°32′ S, 151°40′ E) and in the hinterland of Coffs Harbour, NSW (30°03′ S, 152°59′ E). These localities included four groups of magpies in remote regions where humans would be seen quite rarely and six regions on the outskirts of the town where humans would be seen quite often. All groups of magpies were in territories frequented by raptors but encounters are likely to have been more common in remote localities (e.g., during our experiments within the territories on the outskirts of town we saw raptors on several occasions but less often than in the remote localities).

#### Stimuli presented

The alarm calls of the Australian magpie have been described previously (Kaplan et al., 2009). In these experiments we chose to present four different vocal sequences:

(1) consisted of repeated sequences of the generic alarm call (Fig. 1B), a harsh call consisting of single syllables with a dominant frequency that decreases slightly (mean of 1.94 kHz midway) and harmonics spaced apart by 1 kHz, referred to as the ‘generic alarm call’ because it varies little and is used widely in contexts of low threat.
(2) the “eagle” alarm call, a complex, tonal call with less distinct syllables and undulating frequencies (Fig. 1C), elicited by seeing an eagle (Kaplan et al., 2009),
(3) mixed generic and eagle alarm calls played in random sequence (either the generic or eagle call first) in bouts of equal length, chosen because magpies frequently intersperse generic and eagle alarm calls within one sequence of calling (Kaplan et al., 2009)
  and
(4) the warble, a melodious sequence (here 10 and 20 s bouts).

The calls were recorded from two individual adults belonging to two different and non-adjacent groups (50 km apart) near Armidale. Two versions of the same types of calls from these different locations and individuals were presented in random order to groups of magpies not belonging to any of the groups from which the recordings were taken (i.e., all calls presented were those of strangers). Each call type contained some natural variation that was maintained for a bout of at least 5 s. The playback sequence was of a five-minute duration, edited to contain all variations adding also some repeats and some natural breaks of a few seconds (maximum of 2 s break, as observed in their natural call sequences), and then transcribed to a digital recording on an iPod. Playback in the field was standardised to 70–80 dB measured 10 m from the speaker and delivered via powerful battery driven external stereo speakers. Presentation of a call occurred only once on any single day. The order of presentation was randomised and each type of call was repeated a mean of 5 times (each time on a different day) to each group of magpies (see below about some variation). A test commenced only when the magpies were foraging on the ground in an open, flat area and were within approximately 50 m of the speaker.

#### Behaviour scored

Behaviour was scored for 5 min immediately before presentation of a call commenced, for 5 min during presentation of the call and for 5 min immediately after the presentation. These three periods will be referred to as pre, dur and post, respectively. A focal bird (always an adult) was selected for each test and its behaviour was scored.

The following behaviour was recorded using pen and paper. A behavioural event was recorded using symbols, and without looking down at the paper, each time the behaviour occurred (event recording):

**Figure 2:**
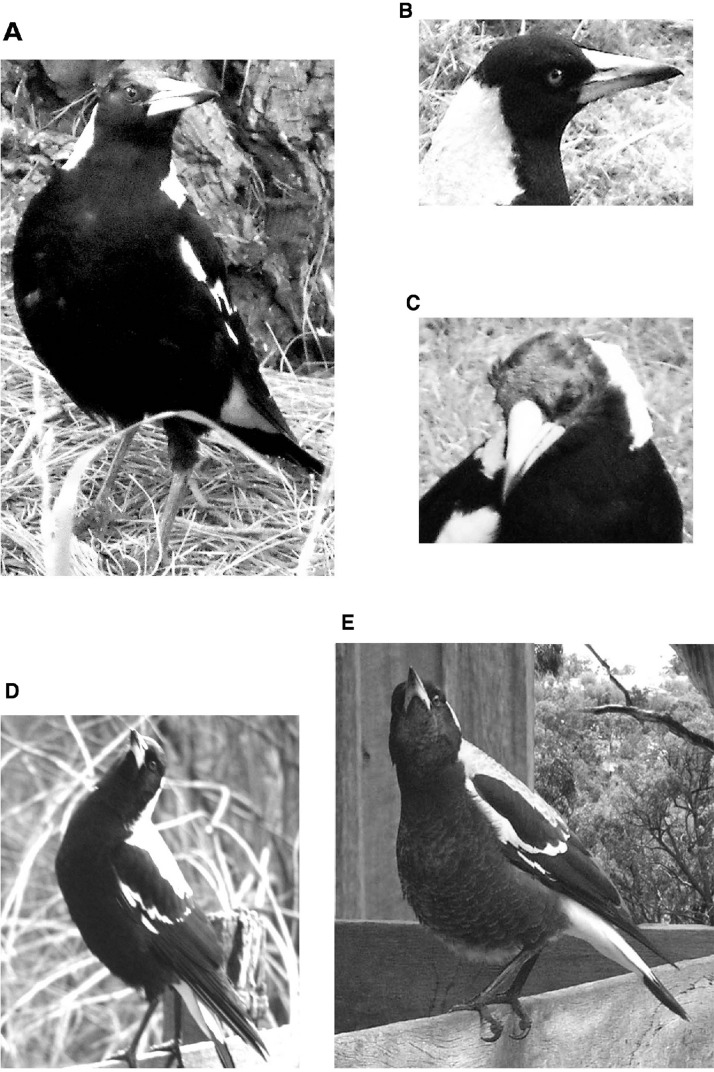
Head positions of the magpies. Illustration of the various head positions recorded. (A) Horizontal scanning, (B) head held horizontally, (C) looking down in this case with no preferred eye, (D) looking up with slight head tilt to look overhead, in this case, with the right eye, (E) looking up without a preferred eye. Note that, although not the case in two of these photographs, all tests were conducted when the magpies were on the ground.

(1) number of events/bouts of look-scan, recorded when the beak was horizontal (parallel to the ground; Figs. 2A and 2B) and head moved from side to side in rapid, jerky movements,
(2) number of events of looking down, beak pointing towards the ground (Fig. 2C), usually prior to pecking (eye choice is often obvious and can be scored),
(3) number of events (bouts) of looking up, recorded when the beak was pointing upwards at an angle at least 30 degrees above horizontal (Figs. 2D and 2E). Looking up could be sustained looking without head movement, often with the head tilted to allow use of one eye to look overhead, or it could involve rapid, small movements back and forth. An additional record was made of which eye was used to look overhead provided that a head tilt was obvious,
(4) foraging, scored as the number of pecks at the ground or at an insect flying just above ground level.

### Experiments conducted in Fiji

The same tests were conducted in Taveuni, Fiji, at the southern-most tip of the island in the region of Vuna and Navakawau (approximately 16°55′ S, 179°55′ E). Our investigations took place during April and May 2006, using the same method as above and presenting the same calls.

Unlike their Australian counterparts, the Taveuni magpies were shy and flighty, often hiding amongst shrubs and palms and the groups were widely dispersed, difficult to spot and follow through rough terrain and often difficult to reach. Also poor weather (strong wind or rain) made it at times impossible to test them on many days. Nonetheless, we were able to identify eight magpie groups (none were bachelor groups) on the island and tested five family groups of 3–5 birds each, similar to the family group sizes tested in Australia. To be comparable with the experiments conducted at our Australian research sites, we adhered to the same requirement for the magpies in Taveuni as in New South Wales: they had to be on the ground, to be together as a group (or at least within visual range) before testing commenced. Despite some of the described difficulties, we managed to run 23 complete playback sessions, achieving at least one repeat for each family (4 tests with the generic alarm call, 6 with the eagle alarm call, 9 with random sequences of these two calls and 4 with the warble).

#### Analysis of data

The data collected in Australia were analysed using SPSS by 3-way ANOVAs using the factors Period (pre, dur, post) as a repeated measure, Call type and Locality. Post hoc tests were Tukeys. We were most interested in main effects of Call type and any significant interactions with Call type. A Bonferroni adjustment was made to set α at 0.017 for the three types of looking scores. There was some variation in the group sizes used in the analysis because scores were not used unless it was possible to score the birds over all three periods of one presentation/trial (e.g., if the birds flew away from the area, a trial was stopped and any records made were disregarded). However, a particular behaviour could be scored as zero in any period of a trial provided that the magpies were present and hence performed other behaviour. Such zero scores were rare. The total number of trials was 202, since it included two extra tests made inadvertently within the protocol.

The data collected in Fiji were analysed using non-parametric statistics because the sample sizes were too small to be certain that they were distributed normally and had equal variance. Wilcoxon tests were applied, using the Bonferroni adjustment as above.

Lateral bias in eye preference was determined using z-scores, using the formula *L*−(*L* + *R*/2)/√(*L* + *R*/4), where *L* is the number of left scores and *R* the number of right scores.

#### Approval and licence

This research was conducted under approval of the Animal Ethics Committee at the University of New England and the Scientific Licence number S10361 issued by National Parks and Wildlife, NSW.

## Results

### Magpies tested in Australia

#### Looking up

Analysis of the data for looking up revealed significant main effects of Period (*F*_2,324_ = 11.331, *p* = 0.000), Call type (*F*_3,162_ = 2.998, *p* = 0.032) and Locality (*F*_9,162_ = 4.704, *p* = 0.000). There were two significant interactions; viz., Period × Call type (*F*_6,324_ = 2.695, *p* = 0.014) and Period × Locality (*F*_18,324_ = 3.147, *p* = 0.000).

**Figure 3:**
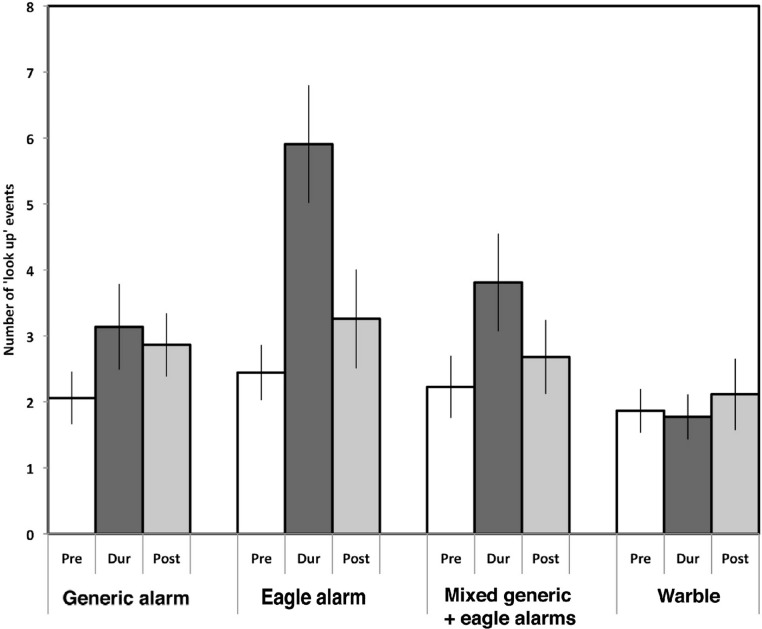
Looking overhead. The mean number of looking up scores (‘look up’ events) recorded in the 5-min periods prior to (pre, white bars), during (dur, dark grey bars) and after (post, light grey bars) presenting the four different calls. Standard errors are indicated. Note the elevated scores during presentation of all but the warble call, and especially during presentation of the eagle alarm call.

Figure 3 presents the mean scores (with standard errors) for Period and Call type. Looking up was elevated during presentation of any of the three alarm calls (*p* ≤ 0.0018 in each case), especially during presentation of the eagle alarm call, but not during presentation of the warble. Tukeys post hoc tests showed that the increase in looking up in response to the eagle alarm call was significantly higher than in response to the warble (*p* = 0.007). None of the other differences between calls were significant (*p* ranged from 0.168 to 0.981).

**Figure 4:**
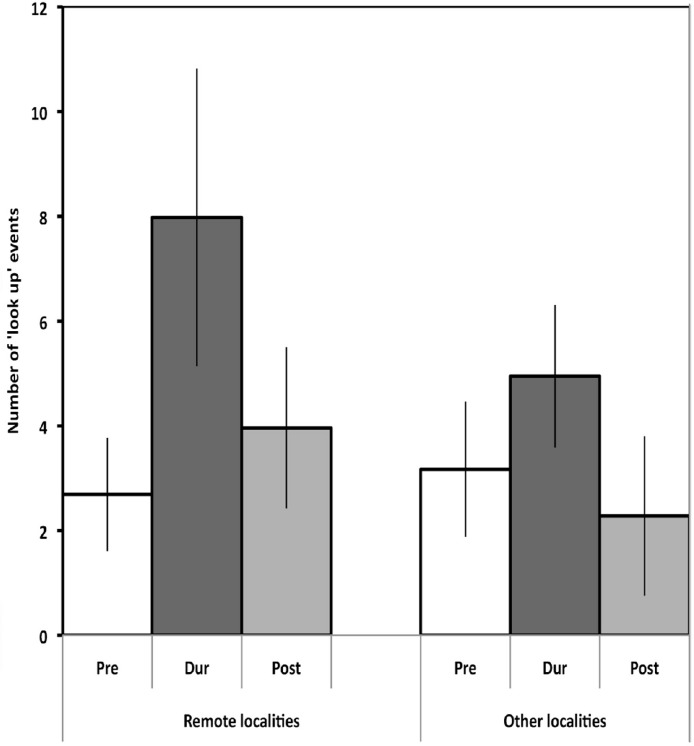
Comparison of responding by magpies in remote and semi-urban localities. The mean number of looking up events in groups of magpies in more remote localities (4 groups) compared to those in localities on the outskirts of town (6 groups labelled other localities in the Figure). Note the higher level of looking up during presentation of the calls (all presentations lumped) in the former group. Pre, Dur and Post labelling is as in Fig. 3.

The significant interaction between Period and Locality was due to some variation between localities in pre scores (i.e., in the 5 min before playback occurred) and in the amount of elevation of scores during playback. Magpies in four of the 10 localities showed greater increases during playback than the rest (*p* < 0.017 in each case) and these four localities were the ones most remote from human habitation (Fig. 4). In fact the strongest response was recorded in the remote hinterland of Coffs Harbour.

It is important to note that there was no interaction between Locality and Call type (Locality × Call type, *F*_27,162_ = 0.635, *p* = 0.917; Locality × Period × Call type, *F*_54,324_ = 0.751, *p* = 0.893). This means that the pattern of increase in looking up during presentation of the alarm calls, and not the warble, occurred generally, across all localities despite some variation in the magnitude of the increase between localities (above).

In only 67 cases of looking up was it possible to record accurately use of the left or right eye to look up during presentation of the alarm calls. Eye preference determined from these observations was 84.3% left eye (Z-score test, *z* = 2.32, *p* < 0.05). Considering each alarm call separately, the eye preference for looking up during the generic alarm call was 75% left, during the eagle alarm call 79% left and during the mixed generic and eagle alarm calls 85% left (all significantly divergent from no eye preference, *p* < 0.05).

Eye preference was recorded in 145 occasions of looking up when no sound was presented and here the eye preference was 47.6% left (*z* = 0.44, no significant preference). In other words, there was no bias to use a preferred eye when no call was being played. The scores of looking up during presentation of the warble were too few to determine eye preference.

#### Look-scan

**Figure 5:**
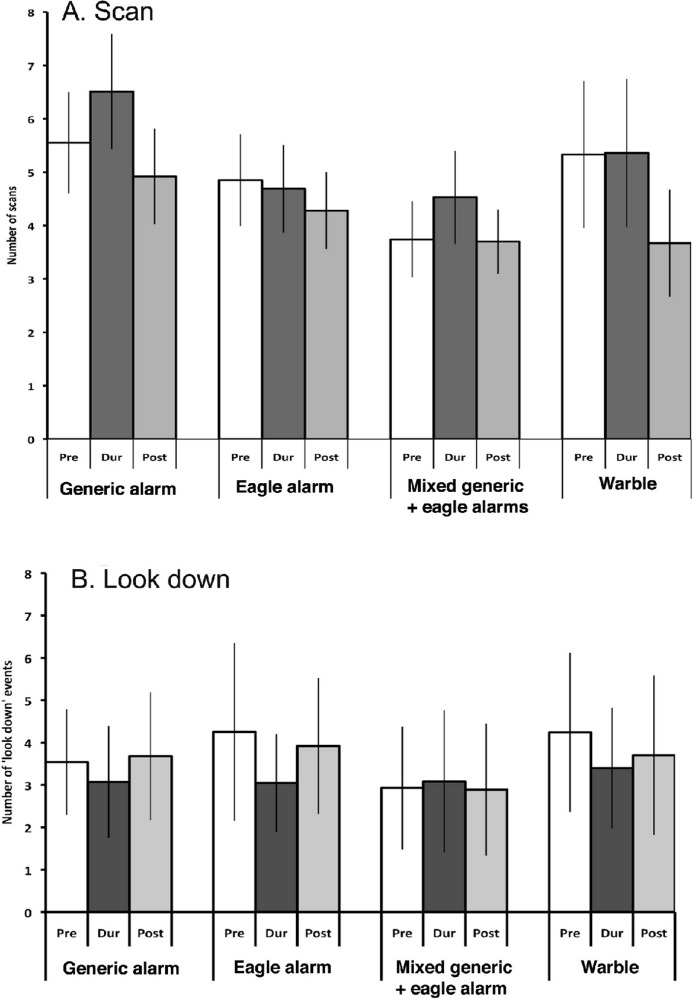
Horizontal scanning and looking down. The data for scanning events with the beak horizontal (parallel to the ground) are presented as in Fig. 3. Note the absence of any significant effects. (A) look scan in the horizontal plane, (B) looking down, with the beak pointed towards the ground.

The scores of look-scan (with beak horizontal) showed significant effects of Period (*F*_2,320_ = 3.415, *p* = 0.034) and Locality (*F*_9,160_ = 16.568, *p* = 0.000) but no significant effect of Call type (*F*_3,160_ = 0.637, *p* = 0.593). In fact, there was also no significant interaction with call type (Period × Call type, *F*_6,320_ = 0.552, *p* = 0.768; Period × Call type × Locality, *F*_54,320_ = 0.933, *p* = 0.610). Overall, horizontal scanning did not change from pre- to during presentation of any of the call types but it tended to decrease slightly in the post-test period (Fig. 5A). It was higher in three localities, irrespective of Period, but there was no pattern that matched the one seen for looking up (i.e., not necessarily higher in the more remote localities). Most important was the absence of any relationship of scanning to call type.

#### Look down

The number of times that the magpies looked down did not vary with Call type (*F*_3,148_ = 0.568, *p* = 0.637) and there was no interaction between Call type and Period (*F*_6,296_ = 1.867, *p* = 0.086). There was, however, a three-way interaction between, Period × Locality × Call type (*F*_54,296_ = 2.279, *p* = 0.000) and an interaction between Period × Locality (*F*_18,296_ = 3.3557, *p* = 0.000). Significant main effects were found for Locality (*F*_9,148_ = 20.37, *p* = 0.000) and Period (*F*_2,296_ = 5.088, *p* = 0.007). In some localities, looking down decreased during presentation of the calls but the pattern was the same for all calls and not related to call type (Fig. 5B).

#### Foraging

Analysis of the scores for foraging events revealed significant main effects of Period (*F*_2,196_ = 7.98, *p* = 0.000), due to a decrease in foraging during presentation of the calls, and Locality (*F*_8,98_ = 3.735, *p* = 0.001), due to variation in the amount of foraging in different localities, but no significant effect of Call type (*F*_3,98_ = 1.284, *p* = 0.284). There were no significant interactions with Call type (Period × Call type, *F*_6,196_ = 1.105, *p* = 0.361; three-way interaction, *F*_36,196_ = 1.253, *p* = 0.169). The only significant interaction was between Period and Locality (*F*_16,196_ = 3.406, *p* = 0.000), largely due to location variation in return to foraging in the post-presentation period.

### Magpies tested in Fiji

We observed that the magpies in Taveuni, Fiji, came to the ground less often than those in Armidale, Australia. The magpies in Fiji were extremely vigilant of avian predators and readily mobbed them and they also used the unusual behaviour of using the beak and body in order to point at a predator for the benefit of the group, a behaviour that has since been described (for the first time in an avian species) and is reported elsewhere (Kaplan, 2011).

**Figure 6:**
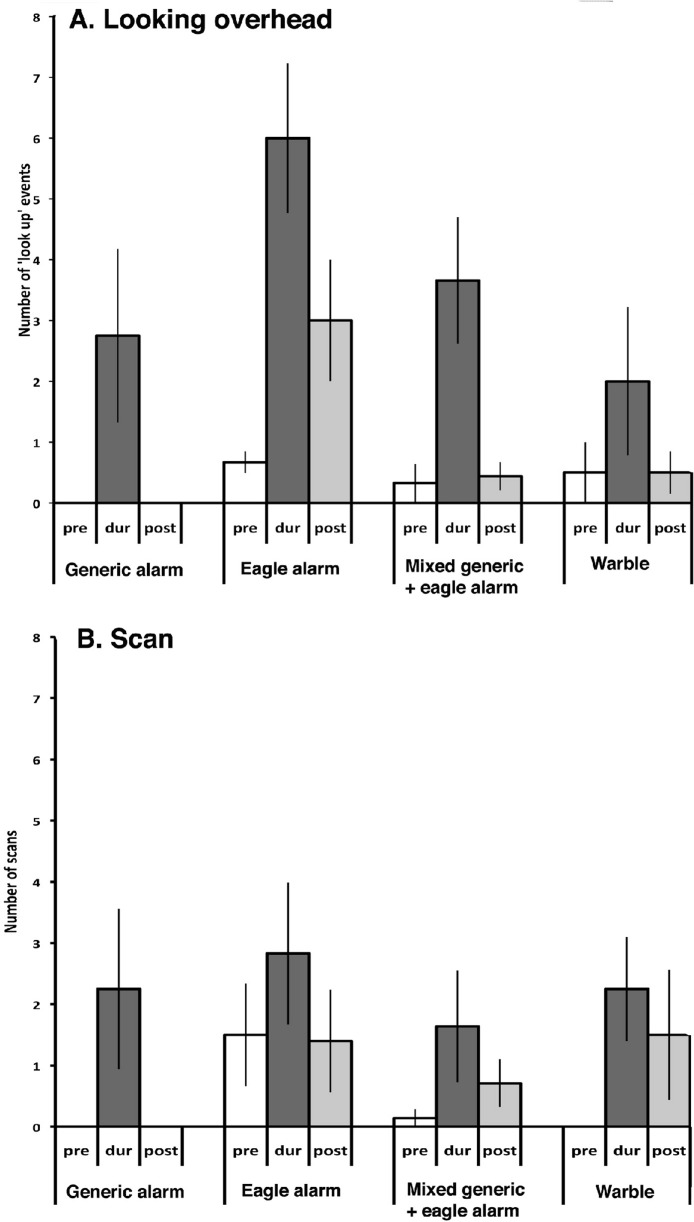
Responses of magpies in Fiji. The results obtained by testing magpies in Taveuni, Fiji, are presented as in Fig. 3. (A) look up, (B) look scan with the beak parallel to the ground. Note in particular the significant elevation of looking up during presentation of the calls, especially during presentation of the eagle alarm call.

#### Looking up

As Fig. 6A shows, looking up was elicited by all of the alarm calls: significant Friedman test (<0.020) was followed by 1-tailed (direction of change predicted) Wilcoxon tests with Bonferroni corrections. It was found that the increase in looking up events from pre- to during presentation was significant for the presentation of the mixed generic and eagle alarm calls (*N* = 9, *T* = 0, *p* < 0.005) and significant or close to significant for the eagle alarm call (*N* = 6, *T* = 0, 0.010 < *p* < 0.025). All four presentations of the generic alarm call led to an increase in looking up but the sample was too small for statistical analysis. There was no significant trend for change in tests in which the warble was presented (increase in 2 tests and no change in the other 2 tests).

Comparisons of the looking up scores during presentation and post-presentation showed a significant decrease in the case of the mixed generic and eagle alarm calls (*N* = 8, *T* = 0, *p* = 0.005) and a trend for the same in tests with the generic alarm call alone but no significant change in tests with the eagle alarm call (looking up remained elevated in the post- compared to the pre-test period) or the warble.

The magpies in Fiji responded by looking up most strongly to the eagle alarm call but 2-tailed U test comparisons (used because the groups did not contain identical individuals) of the scores during the eagle alarm call and the mixed generic and eagle alarm calls revealed only a trend towards a significant difference, *U* = 4.5, 0.05 < *p* < 0.10, and comparison of looks up during the eagle alarm call and the generic alarm call also only approached significance (*U* = 4.5, *p* = 0.071).

The eye used to look up could be recorded in 30 events during presentation of the alarm calls, 22 of which were use of the right eye (73%). This preference to use the right eye is significant (*z* = 3.615, *p* < 0.05) and opposite to the finding of a left eye preference in the birds tested in Australia.

#### Look scan

Although scanning with the beak held horizontal seemed to increase during presentation of the calls (Fig. 6B), comparison of scores in the pre-period and during failed to reveal any significant differences (*p* > 0.10).

#### Look down

Looking down was recorded too rarely to warrant detailed analysis but no pattern of change across the periods pre-, during and post- was apparent, except in the case of the warble: the number of look-down events increased during presentation of the warble in all four tests applied.

## Discussion

The most important finding was a significant increase in looking up (overhead) when the specific alarm calls that we had previously identified as being related to the presence of birds of prey (the generic, eagle and mixed eagle and generic calls) were played. In the Australian population we had thought of the call in Fig. 1C as an eagle alarm call because the results were very consistent, particularly in areas where wedge-tailed eagles (*Aquila audax*) and little eagles (*Hieraetus morphnoides*) are common and both these species are known to kill adult magpies. In Australia, this increase in looking up was greater in magpies in the more remote localities compared to those with territories closer to the urban environment. We consider that the higher incidence of looking up in remote areas was due to the presence in these localities of more avian predators, such as little eagles and wedge-tailed eagles.

The same response to the alarm calls occurred in the magpies tested in Australia and in Fiji. In Fiji, wedge-tailed eagles and little eagles do not occur. Instead, the dominant predators of magpies are goshawks, which particularly seek out magpie juveniles. As mentioned before, we actually witnessed goshawk pursuits of juvenile magpies. Peregrine falcons and even Pacific harriers would be capable of taking adult magpies. It is likely that the need to warn about these aerial predators was an important factor in maintaining use of the alarm signals in the birds that had been translocated to Fiji. Alternatively, it could be argued that the alarm signalling persists after translocation simply because it is a robust trait not influenced to any noticeable degree by relaxation of a need to learn or of environmentally induced changes to gene regulation pathways (Lahti et al., 2009), although we think this is less likely.

In the magpies tested in Australia and Fiji scanning in the horizontal plane did not vary significantly with call type. Looking down was also invariant with respect to call type. This shows that the alarm calls did not signal a general increase in looking but, rather, an increase in looking specifically overhead in the direction of a probable attack. Foraging decreased during presentation of the alarm calls, and so did walking, as might be expected since the birds attended to the calls but these decreases showed no variation related to call type. They occurred in response to hearing any of the alarm calls presented.

The increase in looking up tended to be strongest when the eagle alarm call was played and this was found in both the Australian and Fijian samples. This would suggest that this specific call communicates more information about the threat from an overhead predator than does the generic alarm call. In fact, the eagle alarm call presented alone had the strongest effect, weakened somewhat by mixing the generic and eagle alarm calls. This might be simply a dilution effect (the eagle alarm call being heard only half as often in presentations of the mixed calls). Alternatively, it could mean that the eagle alarm call is more potent when it is heard on its own. In fact, our previous study (Kaplan et al., 2009) showed that eagle alarm calls are made on first sighting an eagle, whereas the generic and other alarm calls are produced somewhat later as swooping behaviour occurs (i.e., once mobbing takes place).

Another explanation for the lesser increase in looking up on hearing the mixed generic and eagle call sequence compared to the eagle call alone could be that the mixed call may have its own specific meaning, similar to findings about the alarm calls of Putty-nosed monkeys, *Ceropithecus nictitans* (Arnold & Zuberbühler, 2008; Arnold & Zuberbühler, 2012; Zuberbühler, 2009), which produce “hack” calls when they see an eagle, “pyow” calls to a leopard and males produce “pyow-hack” sequences when leaving an area. Since the latter elicits following by the group, the combination conveys an entirely different message than either call alone. Although we did not detect a qualitatively different response to the mixed call sequence compared to either the generic or eagle calls alone, the meaning of the combined call may be interpreted by the magpies as at least quantitatively different from either call alone.

Additional evidence in support of the specificity of the alarm calls comes from the significant preference of the magpies in Australia to use their left eye when looking overhead. As explained in the Introduction, this is consistent with findings in other species. Moreover, this left eye preference contrasts with a significant preference, in magpies, to use the right eye (or ear) during foraging (44% left), as we have reported previously (Rogers & Kaplan, 2006). We have also reported previously a strong left eye preference (97% left) to view moving food objects (Rogers & Kaplan, 2006). These eye preferences in magpies are consistent with those found in comprehensive studies of domestic chicks; viz., right eye for discriminating food from background and left eye for spatial cues and responding to predators (summarised in Rogers, 2008). Preferential use of the left eye initiated by hearing alarm calls is added evidence that these calls may have a specific, referential meaning (e.g., “eagle” plus “emergency/escape”).

The right eye preference to look overhead recorded in the magpies tested in Fiji is intriguing because it shows that these magpies used their left hemisphere to locate and identify the potential aerial predator signalled by the alarm calls. As summarised in Rogers (2008), the left hemisphere categorises stimuli and is used to distinguish items from a distracting background. It is conceivable that the magpies in Fiji used their right eye and left hemisphere because they needed to distinguish predators against the background. In fact, they were almost always tested in conditions in which trees/palm fronds were overhead and there was no clear view of the sky, whereas the magpies tested in Australia were always in open areas with a clear view of the sky. As we observed, the raptors in Fiji are usually concealed in the canopy, where they were frequently seen perched ready to swoop at their prey. The opposite eye preference for looking up in magpies in Australia compared to those in Fiji could, therefore, be due to ease of distinguishing a predator against the background. Alternatively, the different eye/hemisphere preference in the magpies in Fiji could be due to a somewhat different interpretation of the alarm calls, perhaps due to a change in the repertoire such that the alarm calls recorded from magpies in Australia and presented in Fiji may have been more difficult to interpret by the magpies in Fiji. The cognitive processes of the left hemisphere may, therefore, be required in preference to the rapid processing of the right hemisphere controlling responses in emergency situations (MacNeilage, Rogers & Vallortigara, 2009; Rogers, Vallortigara & Andrew, 2013). Further research is required to determine which of these explanations may be correct.

In summary, our findings suggest that the alarm calls, the eagle alarm call in particular, are signals with specific meaning or, expressed differently, they are referential signals (Evans, 1997). The most compelling element in our discovery of a referential signal in Australian magpies is the location of the playback apparatus and the subsequent head movement by magpies. The source of the sounds of all playback sequences was a speaker placed on the ground. Yet the magpies responded by looking up and overhead (significantly so) when they heard the eagle alarm call but not when they heard the warbling sequence. Playback of warbling sequences occasionally led to direct investigation of the speaker, i.e., the source of the sound as one would expect a territorial species to do when a foreigner’s song was played back in the middle of their territory. Magpies are known for their highly efficient policing of their territory (Kaplan, 2008). Our tests showed that magpies distinguished between sound source and sound message and they did so consistently. We have shown previously that magpies produce the eagle alarm call when they see a model eagle and now we have evidence in support of our hypothesis that this call communicates to conspecifics the presence of an eagle or, as the Fiji sample indicates, it may be more generally applied to any raptor species of life threatening concern to magpies. As mentioned in the Introduction, it could be considered that response to the alarm calls remained stable in the translocated magpies despite a possible relaxation of selective pressures to retain them in the new environment. However, we think this is unlikely since it seems that predatory pressures are a strong influence on maintaining this particular signal. Indeed, Clunie (1981) has reported “particularly high densities” of the genus *Accipiter* in Taveuni, Fiji, and we definitely gained the impression that Taveuni has a much higher concentration of raptors than in the source country, NSW Australia.

This apparent stability of response to alarm calls has to be seen in the context of the magpies’ vocal plasticity and ongoing flexibility of learning. It is important to note that magpies acquire new syllables throughout their lives. Wherever magpies establish territories, about 25% of their repertoire will eventually overlap with that of neighbours (Farabaugh, Brown & Veltman, 1988). They are also capable of mimicry and acquisition of new sounds as adults, including human speech (Kaplan, 1999). The stability of this signal of an alarm call specifically maintained to communicate the presence of a raptor may, therefore, be all the more remarkable and convincing. It is also noteworthy to add that the eagle alarm call may be learned. It is not part of the general repertoire of magpie nestlings. Kaplan recorded and analysed the vocal repertoire of 45 nestlings and juvenile magpies from different family groups and found no evidence of specialised alarm calls. Juvenile magpies were confined to using distress calls and, very rarely, generic alarm calls by the seventh month post fledging (Kaplan, 2008).

Having identified the circumstances in which magpies look up in response to hearing a recording of their alarm calls, we have added evidence that the eagle alarm call is ‘functionally referential’ (Macedonia & Evans, 1993; Evans, 1997). Moreover, using playbacks in Fiji of the identical vocal sequences that we used in Australia, we have obtained evidence that a referential signal passed on vocally may retain its meaning even in populations that have been isolated for a considerable period of time and over many generations, in this specific case for at least 10 generations since the most recent introduction.

## Additional Information and Declarations

### Field Study Permissions

The following information was supplied relating to ethical approvals (i.e., approving body and any reference numbers): Scientific Licence, National Parks and Wildlife, NSW, Australia, number S10361.
